# Predictive role of microRNA-related genetic polymorphisms in the pathological complete response to neoadjuvant chemoradiotherapy in locally advanced rectal cancer patients

**DOI:** 10.18632/oncotarget.7757

**Published:** 2016-02-26

**Authors:** Eva Dreussi, Salvatore Pucciarelli, Antonino De Paoli, Jerry Polesel, Vincenzo Canzonieri, Marco Agostini, Maria Luisa Friso, Claudio Belluco, Angela Buonadonna, Sara Lonardi, Chiara Zanusso, Elena De Mattia, Giuseppe Toffoli, Erika Cecchin

**Affiliations:** ^1^ Experimental and Clinical Pharmacology, Centro di Riferimento Oncologico, National Cancer Institute, Aviano, Italy; ^2^ Department of Surgical, Oncological and Gastroenterological Sciences, Section of Surgery, University of Padova, Padua, Italy; ^3^ Radiation Oncology, Centro di Riferimento Oncologico, National Cancer Institute, Aviano, Italy; ^4^ Epidemiology and Biostatistics, Centro di Riferimento Oncologico, National Cancer Institute, Aviano, Italy; ^5^ Pathology, Centro di Riferimento Oncologico, National Cancer Institute, Aviano, Italy; ^6^ Nano Inspired Biomedicine Laboratory, Istituto di Ricerca Pediatrica, Città della Speranza, Padua, Italy; ^7^ Department of Nanomedicine, The Methodist Hospital Research Institute, Houston, Texas, USA; ^8^ Radiation Oncology, Istituto Oncologico Veneto, IRCCS, Padova, Italy; ^9^ Surgical Oncology, Centro di Riferimento Oncologico, National Cancer Institute, Aviano, Italy; ^10^ Medical Oncology B, Centro di Riferimento Oncologico, National Cancer Institute, Aviano, Italy; ^11^ Medical Oncology 1, Istituto Oncologico Veneto, IRCCS, Padova, Italy

**Keywords:** microRNA, polymorphisms, rectal cancer, neoadjuvant therapy

## Abstract

In rectal cancer, a pathologic complete response (pCR) to pre-operative treatment is a favourable prognostic marker, but is reported in a minority of the patients. We aimed at identifying microRNA-related host genetic polymorphisms predictive of pCR.

A panel of 114 microRNA-related tagging polymorphisms was selected and analyzed on 265 locally advanced rectal cancer patients treated with neoadjuvant chemo-radiotherapy. Patients were stratified in two subgroups according to the radiotherapy dose (50.4Gy for 202 patients, 55.0Gy for 78 patients). Interactions among genetic and clinical-pathological variants were investigated by recursive partitioning analysis.

Only polymorphisms with a consistent significant effect in the two subgroups of patients were selected as predictive markers of pCR. The results were validated by bootstrap analysis. *SMAD3*-rs744910, *SMAD3*-rs745103, and *TRBP*-rs6088619 were associated to an increased chance of pCR (p=0.0153, p=0.0471, p=0.0125). *DROSHA*-rs10719 and *SMAD3*-rs17228212 had an opposite detrimental effect on pathological tumour response (p=0.0274, p=0.0049). Recursive partitioning analysis highlighted that a longer interval time between the end of radiotherapy and surgery increases the chance of pCR in patients with a specific combination of *SMAD3*-rs744910 and *TRBP*-rs6088619 genotypes.

This study demonstrated that microRNA-related host genetic polymorphisms can predict pCR to neo-adjuvant chemo-radiotherapy, and could be used to personalize the interval time between the end of radiotherapy and surgery.

## INTRODUCTION

Preoperative 5-fluorouracil (5-FU)-based chemoradiotherapy (CRT) or short-course radiotherapy (RT) followed by total mesorectal excision are the standard treatments for patients with locally advanced rectal cancer (LARC) [1-3]. A complete pathological response (pCR) is reported in 7 to 44% of treated patients [4-10].

Patients with a pCR after CRT demonstrated a significantly improved prognosis [11] with implications for an organ preservation strategy either with trans-anal local excision [12-15] or with an observational approach [11, 16, 17]. For non-responding patients, other therapeutic strategies should be considered, without delaying surgery and sparing patients from useless and potentially toxic CRT [18].

A candidate pathway pharmacogenetic approach was adopted in previous studies to identify predictive markers of pathological response in rectal cancer. A predictive role of genetic polymorphisms (SNPs) in folate metabolism [19-22], DNA repair [23] and cell growth [24, 25] pathways was pointed out.

More recently, SNPs in genomic regions associated to microRNAs function were demonstrated to have higher probability to be expression quantitative trait loci as compared to SNPs in all the other genomic regions. miRNA-related SNPs are located in miRNA-encoding genes, in their mRNA target regions, or in genes involved in miRNA transcription/maturation. A single miRNA-related SNP can have a downstream down-regulation effect on a large number of genes [26]. A broad suppression of genes involved in DNA repair, angiogenesis, and inflammation, has the potential to affect patients response to RT [27].

In this study, we analyzed a panel of 114 miRNA-related SNPs in 265 homogeneous LARC patients treated with neoadjuvant 5-fluorouracil (5-FU)-based CRT with two different RT dose levels of 50.4Gy or 55.0Gy. The primary aim was the identification of RT-dose-independent genetic markers of pCR to neoadjuvant CRT. Secondary aim consisted in defining the interactions of the predictive SNPs with the patients’ clinical-pathological parameters.

## RESULTS

### Patients and genotyping

Patients clinical and pathological data (age, gender, clinical tumour stage -cT-, clinical nodal and metastasis stage -cN and cM, respectively-, tumour distance from anal margin, neoadjuvant treatment parameters -RT dosage, fluoropyrimidines administration, concomitant platinum administration-, date of diagnosis, tumour regression grade -TRG-, surgery, end of RT, post-CRT pathologic T stage -ypT-, kind of surgical intervention, IORT, recurrence, adjuvant treatment, date of last follow-up/death) were collected from the medical records. Patients characteristics according to RT dosage are reported in Table 1.

**Table 1:** Distribution of patients, according to treatment (radiation therapy dose) and clinical features

	RT 50.4Gy(*n = 188)		RT 55.0Gy(*n* = 77)		χ2
	*N*	(%)	*N*	(%)	
Sex					
Man	55	(29.3)	28	(36.4)	
Woman	133	(70.7)	49	(63.6)	*p* = 0.2573
Age (years)					
<55	47	(25.0)	19	(24.7)	
55-59	37	(19.7)	13	(16.9)	
60-64	35	(18.6)	20	(26.0)	
65-69	36	(19.2)	12	(15.6)	
≥70	33	(17.6)	13	(16.8)	*p* = 0.7264
Distance from anal margin (cm)					
<8	124	(66.0)	54	(70.1)	
≥8	64	(34.0)	23	(29.9)	*p* = 0.5114
Time from surgery to radiation therapy (days)					
<50	67	(38.5)	16	(21.3)	
50-56	43	(24.7)	13	(17.3)	
57-63	27	(15.5)	23	(30.7)	
≥64	37	(21.3)	23	(30.7)	*p* = 0.0032
Unk	14		2		
TRG					
1	53	(28.2)	20	(26.0)	
2	36	(19.2)	20	(26.0)	
3	58	(30.9)	29	(37.7)	
4-5	41	(21.8)	8	(10.4)	*p* = 0.1153

TRG, Tumour Regression Grade; RT, radiotherapy; Gy, Gray; unk, unknown

* 93 out of 188 patients (49.5%) received oxaliplatin in addition to fluoropyrimidines

Disease free survival (DFS) was significantly better among complete responders (TRG = 1) than non-complete responders (TRG = 2-5) (log-rank test *p* = 0.0260) (Figure 2).

**Figure 1:**
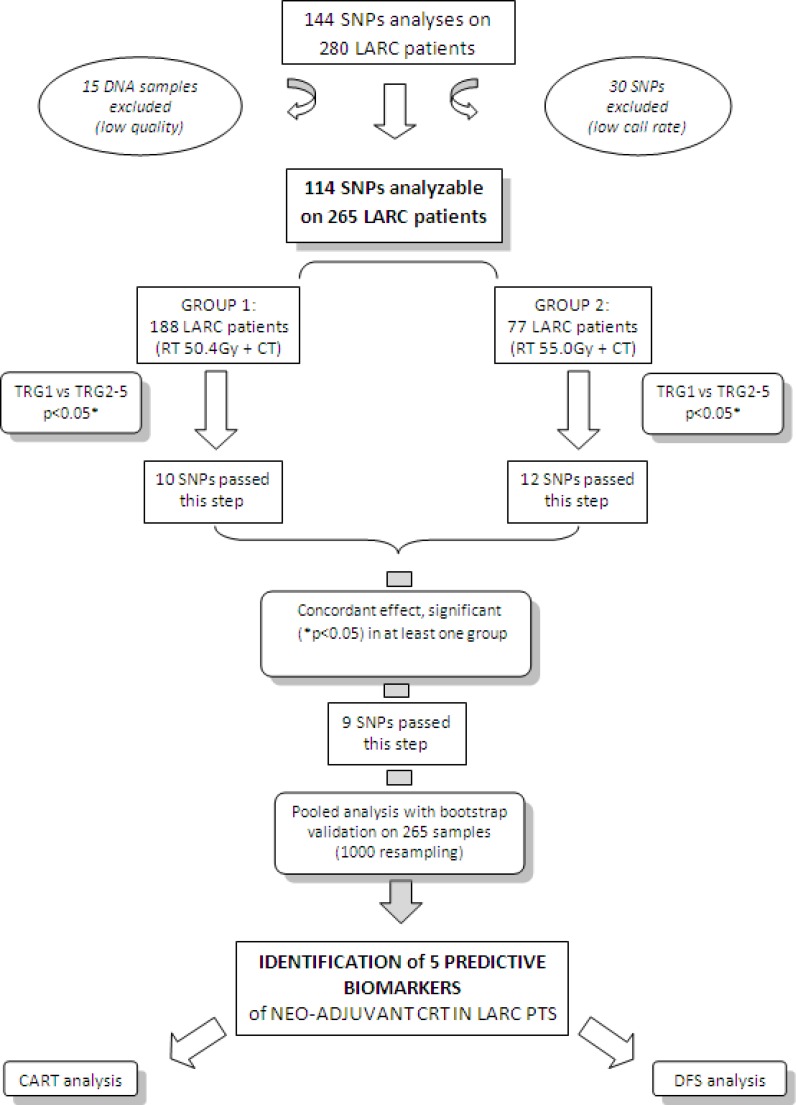
Flow chart of the study LARC, Locally Advanced Rectal Cancer; SNPs, Single Nucleotide Polymorphisms; TRG, Tumour Regression Grade; RT, radiotherapy; CT, chemotherapy; Gy, Gray; CRT, Chemo-Radiotherapy; Pts, patients; CART, Classification And Regression Tree; DFS, Disease Free Survival. *Fisher exact test

**Figure 2:**
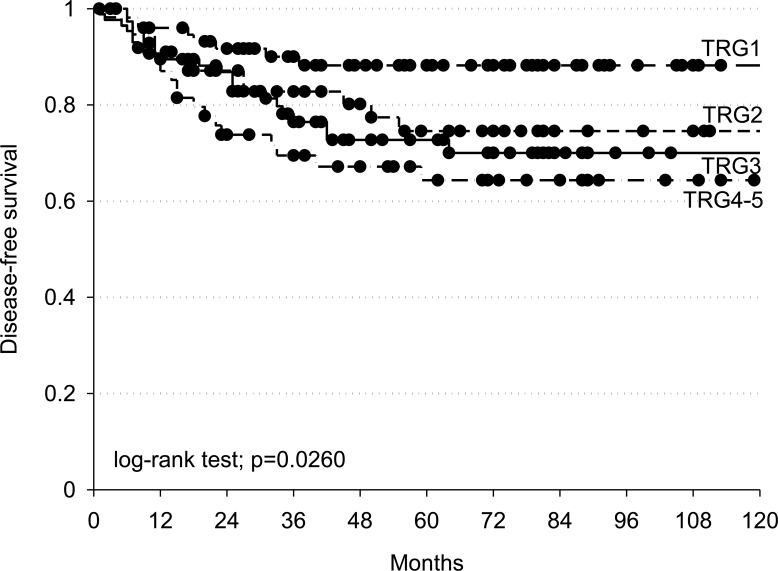
Kaplan-Meier estimates of disease-free survival according to TRG (Tumour Regression Grade) Black dots represent censored patients.

Genotyping analyses were successful in 114 assays out of 144. Fifteen DNA samples were excluded from genotyping due to their poor quality. The average genotype call rate was 98.4% (range: 95.8-100.0%). Three random SNPs were selected for BeadXpress analytical validation by Sanger sequencing. In particular, 93 samples were sequenced for rs17228212, 62 for rs744910, and 99 for rs3823994. All these SNPs had a high concordance rate (100.00% *SMAD3*-rs17228212, 100.00% *Tudor-SND1*-rs3823994, and 98.38% *SMAD3-*rs744910).

### Association of SNPs with pCR in the 2 groups of patients

The association between genotypes and pCR was tested separately in the 2 subgroups of patients treated at different RT dose, with multivariate analysis (Table 2). Ten SNPs resulted significant (*p* ≤ 0.05) in the 50.4Gy group, twelve in the 55.0Gy group. Nine SNPs (*DROSHA*-rs10719, *TRBP*-rs6088619, *SMAD3-*rs17228212, *SMAD3-*rs744910, *SMAD3-*rs745103, *SMAD5-*rs1057898, *SMAD5-*rs6871224, *TNRC6A*-rs6497759, *miR-371a-*rs28461391) resulted significant in at least one group, showed a concordant genetic effect, and compatible genetic models in the 2 subgroups.

**Table 2:** Association between SNPs and pathological complete response (TRG2-5 vs TRG1), according to RT dose

			RT 50.4 Gy (*n* = 188)			RT 55.0 Gy (*n* = 77)		
Gene	SNP	Base change	GM	OR (95% CI)^a^	*p*-value	GM	OR (95% CI)^b^	*p*-value
CNOT4	rs11772832	A>G	R	1.65 (0.65-4.23)	0.2937	R	**0.11 (0.01-0.96)**	**0.0460**
CNOT6	rs6877400	A>G	R	0.28 (0.02-4.83)	0.3785	D	**0.16 (0.03-0.84)**	**0.0297**
DDX20	rs197412	A>G	**A**	**1.83 (1.05-3.21)**	**0.0339**	D	0.73 (0.20-2.70)	0.6401
DGCR8	rs417309	G>A	D	1.73 (0.54-5.59)	0.3553	D	**0.20 (0.04-0.95)**	**0.0428**
DICER1	rs1057035	A>G	**D**	**2.25 (1.07-4.72)**	**0.0327**	R	0.62 (0.12-3.34)	0.5458
DROSHA	rs10719	C>T	**A**	**2.39 (1.24-4.61)**	**0.0094**	R	2.74 (0.21-35.70)	0.4412
TRBP	rs6088619	A>G	**A**	**0.34 (0.15-0.75)**	**0.0073**	D	**0.21 (0.05-0.82)**	**0.0251**
SMAD2	rs1792671	G>A	**D**	**0.16 (0.04-0.63)**	**0.0087**	R	0.29 (0.01-18.05)	0.5540
SMAD3	rs17228212	T>C	**A**	**1.83 (1.02-3.30)**	**0.0446**	A	**3.61 (1.17-11.19)**	**0.0261**
SMAD3	rs2289791	C>A	**A**	**0.58 (0.35-0.97)**	**0.0364**	R	4.60 (0.31-67.46)	0.2657
SMAD3	rs744910	A>G	R	0.50 (0.23-1.07)	0.0739	R	**0.16 (0.04-0.75)**	**0.0201**
SMAD3	rs745103	A>G	R	0.59 (0.27-1.27)	0.1819	A	**0.23 (0.08-0.68)**	**0.0080**
SMAD3	rs8025774	G>A	**D**	**0.47 (0.24-0.92)**	**0.0279**	R	4.33 (0.30-62.78)	0.2824
SMAD3	rs8028147	G>A	D	1.60 (0.83-3.11)	0.1609	A	**0.31 (0.10-0.97)**	**0.0445**
SMAD5	rs1057898	A>G	D	0.71 (0.36-1.39)	0.3157	D	**0.12 (0.02-0.75)**	**0.0238**
SMAD5	rs6871224	A>G	D	0.72 (0.37-1.43)	0.3485	D	**0.10 (0.02-0.61)**	**0.0123**
TNRC6A	rs6497759	T>A	D	1.33 (0.65-2.73)	0.4368	D	**6.63 (1.01-43.48)**	**0.0487**
TNRC6B	rs139911	T>C	**A**	**1.71 (1.04-2.80)**	**0.0353**	D	0.12 (0.01-1.10)	0.0604
miR196A2	rs11614913	C>T	**R**	**0.29 (0.11-0.78)**	**0.0138**	D	0.44 (0.12-1.58)	0.2080
miR371A	rs28461391	C>T	D	0.92 (0.41-2.04)	0.8288	D/A	**0.20 (0.05-0.88)**	**0.0334**

Only associations that are significant (*p* < 0.05) in at least one group of patients are reported. Statistically significant associations are in bold.

a Adjusted for gender, age, distance from anal margin, platinum treatment, and time between radiation therapy and surgery.

b Adjusted for gender, age, distance from anal margin, and time between radiation therapy and surgery

SNPs, single nucleotide polymorphisms; TRG, Tumour Regression Grade; RT, radiotherapy; Gy, Gray; OR, Odds Ratio; CI, Confidence Interval; GM, genetic model; R, recessive; A, additive; D, dominant.

### Association of SNPs with pCR in the pooled population of patients

Considering the concordant, RT-dose independent effect of the previously identified 9 SNPs, we performed a pooled analysis of the combined datasets to increase the statistical power, and then we internally validated the results by bootstrap analysis (Table 3).

**Table 3:** Association between SNPs (with a concordant effect in the 2 groups according to the study criteria) and pathological complete response (TRG2-5 vs TRG1), in the pooled population of patients (*n* = 265)

Genes	SNP	Base change	Genotype frequency						GM	OR (95% CI)^a^	*p*-value	Bootstrap *p*-value
			TRG 1			TRG 2-5						
			AA	Aa	aa	AA	Aa	aa				
**DROSHA**	**rs10719**	**C>T**	0.616	0.370	0.014	0.492	0.450	0.058	A	1.87 (1.10-3.17)	0.0207	**0.0274**
**TRBP**	**rs6088619**	**A>G**	0.747	0.239	0.014	0.863	0.137	0.000	A	0.39 (0.19-0.79)	0.0089	**0.0125**
**SMAD3**	**rs17228212**	**T>C**	0.708	0.264	0.028	0.524	0.377	0.100	A	2.01 (1.22-3.31)	0.0064	**0.0049**
**SMAD3**	**rs744910**	**A>G**	0.219	0.438	0.343	0.289	0.529	0.185	R	0.45 (0.24-0.85)	0.0135	**0.0153**
**SMAD3**	**rs745103**	**A>G**	0.219	0.493	0.288	0.277	0.559	0.165	R	0.48 (0.25-0.94)	0.0316	**0.0471**
SMAD5	rs1057898	A>G	0.366	0.507	0.127	0.500	0.385	0.115	D	0.61 (0.34-1.09)	0.0924	0.0922
SMAD5	rs6871224	A>G	0.366	0.507	0.127	0.497	0.392	0.111	D	0.61 (0.35-1.09)	0.0966	0.0883
TNRC6A	rs6497759	T>A	0.754	0.217	0.029	0.649	0.309	0.042	D	1.70 (0.89-3.22)	0.1076	0.1508
miR371A	rs28461391	C>T	0.718	0.268	0.014	0.783	0.201	0.016	D	0.66 (0.34-1.27)	0.2118	0.2365

Significant associations are reported in bold (*p* < 0.05)

a Adjusted for gender, age, distance from anal margin, time between radiation therapy and surgery, platinum treatment, and total dose of radiation therapy.

SNPs, single nucleotide polymorphism; TRG, Tumour Regression Grade; RT, radiotherapy; Gy, Gray; OR, Odds Ratio; CI, Confidence Interval; GM, genetic model; R, recessive; A, additive; D, dominant.

Five SNPs were still significantly associated with pCR (Table 3). *DROSHA*-rs10719 and *SMAD3-*rs17228212 were associated with an higher risk to be non-complete responders (TRG = 2-5) according to an additive model (OR = 1.87, 95%CI = 1.10-3.17, *p* = 0.0274; and OR = 2.01, 95%CI = 1.22-3.31, *p* = 0.0049 respectively). The same effect was observed for *SMAD3-*rs744910 and *SMAD3-*rs745103, according to a recessive model (OR = 0.45, 95%CI = 0.24-0.85, *p* = 0.0153 and OR = 0.48, 95%CI = 0.25-0.94, *p* = 0.0471, respectively). *TRBP*-rs6088619 showed an opposite effect, according to an additive model (OR = 0.39, 95%CI = 0.19-0.79, *p* = 0.0125).

**Figure 3:**
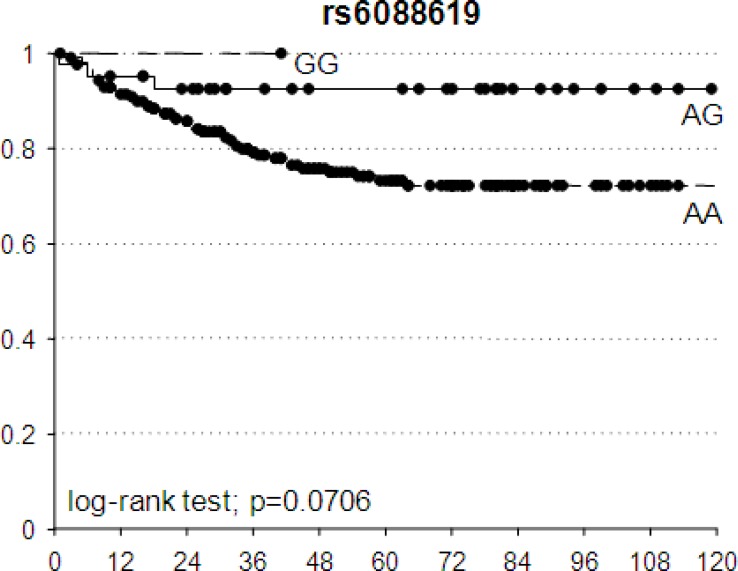
Kaplan-Meier estimates of disease-free survival according to *TRBP*-rs6088619 Black dots represent censored patients.

The potential prognostic value of these 5 SNPs in terms of DFS was investigated. The best fitting genetic models identified by the pCR prediction analyses were used also to compute the HR. An association trend was observed between *TRBP-*rs6088619 and DFS: patients with at least one G allele had a lower probability to have a bad DFS according to an additive model (HR = 0.24, 95%CI = 0.07-0.79, *p* = 0.0706) (Figure 3).

### Association of pCR with clinical-pathological characteristics and SNPs by recursive partitioning analysis

**Figure 4:**
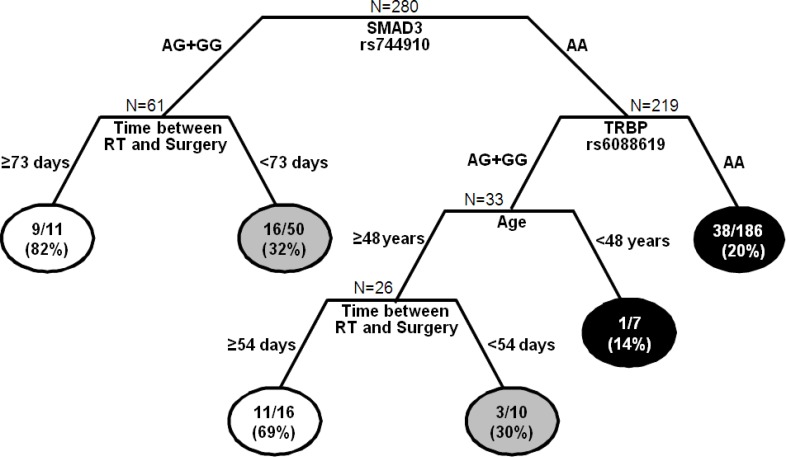
Classification and regression tree of SNPs and clinical-demographic characteristics predictive of pathological complete response (pCR)(TRG1) Terminal nodes report the number and fraction of patients who achieved pCR. White circles represent terminal nodes with high probability of achieving pCR; grey circles represent terminal nodes with intermediate probability of achieving pCR; black circles represent terminal nodes with low probability of achieving pCR.RT, radiotherapy.

To better define the role of SNPs and clinical-pathological features in the response to neoadjuvant treatment, the 5 significant SNPs and some clinical-pathological features (gender, age, RT dose, kind of neoadjuvant treatment, time between the end of CRT and surgery, distance of the tumour from the anal margin) were tested in the CART analysis. As reported in Figure 4, each terminal node identifies a specific combination of genetic and clinical-pathological features that is associated with a different probability to completely respond to neoadjuvant treatment.

The first node is determined by *SMAD3-*rs744910. According to the recessive model, patients were split in those carrying at least one variant allele (AG+GG) and those homozygous for the wild type allele (AA). The first group was further stratified according to the time between the end of CRT and surgery. The longest interval (≥ 73 days) was correlated to the highest probability of pCR (82%). If the interval is shorter than 73 days, the probability to be good responders decreased (32%). Among patients with *SMAD3-*rs744910 AA genotype, *TRBP-*rs6088619 AA genotype was associated with a very low probability to be good responders (20%). For patients carrying at least one G allele, age acted as discriminator. Young patients (< 48 years) were associated with a low probability of pCR (14%). In patients with ≥ 48 years of age, the time between the end of CRT and surgery discriminated complete and non-complete responders.

This analysis highlighted 2 subgroups of patients associated with high probability to achieve a pCR (82% and 69%), 2 with an intermediate probability (32% and 30%), and 2 with low pCR probability (14% and 20%), according to their genetic- and clinical-pathological features.

## DISCUSSION

Up to date no general consensus has been reached about the proper CRT schedule or patients selection for neoadjuvant LARC treatment [2, 3]. Predictive biomarkers of pathological tumour response to pre-operative treatment would be helpful to perform a rational patients selection.

Aim of this study was the identification of miRNA-related SNPs predictive of pathological response to neoadjuvant CRT. We considered a pCR as the clinical end-point of the study, since, consistently with our data (Figure 2), this represents a very reliable prognostic factor nowadays in these patients.

Our major finding was the identification of 3 SNPs in *SMAD3* (rs17228212, rs744910, and rs745103) located in 3 different haploblocks, along with a SNP in *DROSHA* (rs10719), and one in *TRBP* (rs6088619), predictive of pCR. The association between the SNPs and pCR was consistent across two different RT dose levels (50.4Gy or 55.0Gy) administered to the patients, and the results were internally validated by the bootstrap re-sampling technique.

The finding of 3 independent SNPs predictive of pCR in *SMAD3* provides a strong rational to an involvement of this gene in the response to CRT. Consistently with our data, the overexpression of phosphorylated SMAD3 in pre-CRT cancer tissues, was a marker of poor pathological response to fluoropyrimidines-based neoadjuvant CRT in 86 LARC patients [28].

SMAD3 and DROSHA cooperate in miRNA maturation. After being activated by cytokines like TGFβ, SMAD3 directly binds to specific pri-miRNAs promoting their processing by DROSHA complex [29-31]. Pri-miR-21 is among the SMAD3 target pri-miRNAs and its level was previously associated to tumor response to 5-FU-based treatment in patients affected by colon cancer. Specifically, high miR-21 expression in tumours was associated with a poor response to therapy [32]. SMAD2/3 complex can also directly induce the transcription of some miRNAs, like miR-192 and miR-451 [33, 34], previously highlighted as markers of CRT response in different tumor models [35-37].

SMAD3 is also a transcriptional downstream effector in the TGFβ pathway [38], and is involved in inflammation and response to the oxidative stress. TGFβ activates, *via* SMAD2/3 complex, the transcription of *NADPH oxidase 1* and *NADPH oxidase 4*, up-regulating the production of reactive oxygen species, thus potentially enhancing RT efficacy [39].

Drosha is also a downstream effector of proteins as ATM and BRCA1, that are recruited by DNA damage events [40, 41]. Drosha is thus activated after DNA damage, to process another class of non-coding RNAs, called DNA damage response RNAs (DDRNAs), further contributing to DNA repair [42].

The impact of *SMAD3*-rs17228212, -rs744910, and -rs745103 on the encoded protein functionality is mostly unknown, and they were only sporadically studied in the context of clinical trials [43]. No function is predicted for these SNPs by common on-line tools (i.e. snpinfo.niehs.nih.gov) up to date. However, large bioinformatic projects like Encode (http://www.genome.gov/encode/) pointed out the importance of non-coding regions of DNA in determining the gene expression level, that could be under-estimated up to date [44].

*Drosha*-rs10719 is located in the 3′UTR of the gene and, according to SNPinfo web server, can disrupt the binding site of Drosha with miR-181b and consequently decrease Drosha mRNA stability or translation into protein.

*TRBP*-rs6088619 was also identified as predictive marker of pCR in this study. TRBP is a dsRNA-binding protein involved in RISC assembly and Dicer activity. It has been poorly studied up to date in the context of tumour response to CRT. It is a downstream effector of MAPK ERK [45], what activation has been associated with an improved pathological response to fluoropyrimidines-based CRT in rectal cancer [46]. *TRBP-*rs6088619 is an intronic SNP, and at our knowledge its effect on the protein phenotype is still unknown.

In an attempt to study how *SMAD3*, *Drosha*, and *TRBP* genetic variants interact with patients clinical-pathological characteristics we performed a CART analysis, that highlighted *SMAD3*-rs744910 as the most powerful factor discriminating between complete and poor responders.

Moreover, in a genetically defined subgroup of patients, younger people (< 48 years) appeared to have an higher risk of bad response to therapy. This could be explained by the aggressive nature of cancers with an early onset [47].

A noteworthy differential effect of an “actionable” clinical variant, such as the interval between end of CRT and surgery, was highlighted in two different subgroups of patients. At present, a possible improving effect of prolonging this interval on patients response and prognosis was obtained [48, 49]. However, there is no consensus about the proper timing for surgery after RT and, according to our results, patients genetic profile could be considered.

This study is limited by the lack of an independent validation set of patients, and must be considered as explorative and hypotheses generating. However, we performed two independent analyses in two subgroups of patients treated with a different RT dose and we selected only SNPs presenting a dose-independent concordant effect. From a statistical point of view, this strategy lowers the chance of false positive discoveries. Moreover, in an attempt to perform an internal validation, we have applied a bootstrap re-sampling strategy to further select robust predictive markers. In our study the predictive effect of the genetic markers on pCR translated in a non-significant difference in term of DFS. We could hypothesize that other clinical and molecular variables could cooperate with TRG in determining the patients prognosis. In the present study, at pathologic examination of the surgical specimen, pCR was observed in 27.5% of the cases. This value is in the higher range of reported complete pathological response rates, and could be explained by the use in our patients of factors shown to be associated with ypCR such as 50.4Gy or higher, continuous infusion of 5-FU, and two drugs regimens [50].

It is likely that the tumor response to CRT is a complex phenotype with a biological basis that probably depends on a plethora of tumor and host factors. Several previous studies tried to address the issue of pathological response to pre-operative treatment in rectal cancer. Both molecular [51, 52] and radiological [53, 54] measures have been evaluated, but none of them provided a reliable marker to be introduced by itself as selection criteria for patients treatment [55]. Probably only a multi-parameters predictor will definitely address the issue of pCR in rectal cancer. In this context host genetic characteristics must be considered as one of the key players.

In conclusion, we have pointed out in the present study five host genetic markers to be considered for pCR prediction. Three of these markers are located in *SMAD3*, one in *Drosha*, and one in *TRBP*. These factors strictly cooperate in miRNAs processing and clusterize in cellular pathways highly relevant for RT response, as DNA repair and oxidative stress. We have also demonstrated that the interaction of some of these genetic variants with patients age could define specific subgroups for which a longer RT-surgery interval could be suggested. We demonstrated the importance of considering miRNA-related SNPs to identify patients more likely to get a favourable response to neoadjuvant CRT in LARC, that could be redirected to innovative treatment approaches, including the use of more conservative surgical procedures.

## MATERIALS AND METHODS

### Patients and study design

From December 1993 to July 2011, 280 patients were enrolled by CRO-Aviano National Cancer Institute and by IOV-IRCCS and Department of Surgical, Oncological and Gastroenterological Sciences, University of Padova, Northern Italy. Eligibility criteria were the following: histologically confirmed diagnosis of primary resectable LARC, confirmed absence of distant metastases, age ≥ 18 years, Caucasian ethnicity, stage of disease T3-T4 and N0-2, performance status (World Health Organization) 0-2, normal bone marrow-, renal-, and liver function. The neoadjuvant CRT was based on fluoropyrimidines (either 5-FU or capecitabine) with or without oxaliplatin, combined with a dose of 50.4Gy or 55.0Gy of RT. All procedures were reviewed and approved by the Ethical Committee of each participating institution, and all patients signed a written informed consent for research purposes.

The study design is summarized in Figure 1.

### Tumour treatment, response evaluation and follow up

Patients underwent external beam RT with a 10-18MV linear accelerator. A 3D-CRT was used in all patients. Patients were treated in prone position with full-bladder. A dedicated up-down table was used for patient immobilisation and small bowel dislocation outside the target volume, as previously reported [56]. The primary tumour, the mesorectum, the posterior wall of the bladder and prostate/vagina, and the internal iliac nodes represented the clinical target volume (CTV). Patients underwent two different RT programs, according to clinical trials ongoing in the considered period time: 202 patients were treated with a standard dose of 50.4Gy/28 fractions, whereas 78 with a dose of 55.0Gy/25 fractions. In the first group, a consecutive boost of 50.4Gy/3 fractions to the tumour and mesorectum was given following the CTV dose of 45Gy/25 fractions, for a total dose of 50.4Gy. In the second group, a concomitant boost of 10Gy/10 fractions over 5 weeks, 2 times a week (1Gy/fraction, 6 hours interval between the two daily fractions), was delivered to the tumour and mesorectum during the CTV dose of 45Gy fractions, for a total dose of 55Gy. Fluoropyrimidines alone (5-FU 225mg/m^2^/day *iv* continuous infusion for 5 weeks or capecitabine 1650mg/m^2^ in two daily oral administrations for 5 weeks) was prescribed with 50.4Gy or 55.0Gy, whereas the capecitabine (1300mg/m^2^) was administered with oxaliplatin (130mg/m^2^ every 19 days) and concurrently standards RT dose of 50.4Gy.

Standard pathological tumour staging of the resected specimens was performed in accordance with the guidelines of the American Joint Committee on Cancer. Treatment efficacy was defined as TRG [57], and assessed as previously described [58]. All patients were followed-up every 3 months for the first 2 years, every 6 months thereafter up to 5 years, and then yearly.

### SNPs selection

A set of miRNA-related SNPs potentially impacting miRNA maturation and activity was selected.

Patrocles [59] and PubMed (www.pubmed.org) websites were used to identify genes involved in miRNA maturation. A set of genes encoding for miRNAs was included in the analysis. The miRNAs were selected based on their predicted interaction with POLR2A, Drosha, DGCR8, and Dicer (factors involved in miRNA maturation). Only miRNAs predicted by at least three of the considered algorithms (TargetScanHuman-www.targetscan.org-, Microcosm-www.ebi.ac.uk/enright-srv/microcosm/htdocs/targets/v5/-, miRanda-www.microrna.org/microrna/home.do-, Pictar-pictar.mdc-berlin.de-, PolymiRTS-compbio.uthsc.edu/miRSNP-, microSNiper-epicenter.ie-freiburg.mpg.de/services/microsniper-) were selected. The final list of the 63 candidate genes was submitted to Illumina. The Illumina assay design tool (www.illumina.com) identified 13,067 SNPs located in these genes. The SNPs list was further revised according to: the predicted final score (which predicts the quality of the assay giving a score ranging from 0 to 1, cut-off value 0.6), the designability (which ensures the capability to design the selected assay giving a score spanning from 0 to 1, requested value = 1), and the reported minor allele frequency (MAF, cut-off value 5%) in Caucasian population (HapMap CEU). SNPs selection was performed to obtain a good coverage of each haploblock for each gene (according to GenomeVariationServer tool). We finally get a set of 144 miRNA-related SNPs in 51 genes involved in miRNA activity and maturation. In particular, 117 SNPs were localized in miR-machinery factors, and 27 SNPs were located in miRNAs encoding genes (Supplementary Figure 1).

### SNP genotyping

Genomic DNA of LARC patients was extracted from peripheral blood samples using the automated extractor BioRobot EZ1, in association with the Kit “EZ1 DNA Blood Kit 350μl” (Qiagen SPA, Milano, Italy) and stored at +4°C until the time of this study.

The selected 144 SNPs were analyzed using the Illumina BeadXpress platform, that is based on Golden Gate technology. The VeraScan software (version 2.0) was applied for fluorescence detection. GenomeStudio software 2010 (Illumina Inc.) was applied for genotype clustering, with a SNP call-threshold of 0.25 (on a scale of 0-1). Clusters were visually inspected and manually reviewed to ensure high quality data. The control dashboard was checked to evaluate the overall quality of the performed analyses and to exclude samples with low quality. Negative and positive controls were included.

Regions containing the SNPs of interest were amplified using the PCR primers designed according to Primer3Plus (http://www.bioinformatics.nl/cgi-bin/primer3plus/primer3plus.cgi/). Dye-terminator cycle sequencing was performed using the BigDye terminator v3.1 cycle sequencing kit (ThermoFisher Scientific). The amplicons were run on an ABI PRISM 3130xl Genetic Analyzer (ThermoFisher Scientific) and the results were analyzed with Gene Scan software (ThermoFisher Scientific). Primers and PCR conditions are available upon request.

### Statistics

Pathological tumour response to neoadjuvant treatment was defined according to TRG. Complete responders (TRG1) were compared to non-complete responders (TRG = 2-5). RT dose-dependent effects were overcome stratifying patients into 2 groups according to RT dose level (either 50.4Gy or 55.0Gy). A χ^2^ test was applied to evaluate the differences in the distributions of demographic and clinical variables and treatment-related factors between the 2 groups.

The association between genotypes and TRG was tested separately in the 2 groups of patients. Odds ratio (ORs) and 95% confidence interval (95%CI) were computed through logistic regression model, adjusting for gender, age, distance of the tumour from the anal margin, platinum treatment, and time between the end of RT and surgery. Dominant, recessive, and additive genetic models were considered for each genotype combining heterozygous and homozygous genotypes. The best fitting genetic model was selected according to the Wald χ^2^-test. SNPs resulting significant in at least one group, showing a concordant genetic effect, and with a “compatible” genetic model in the 2 groups were further investigated in the entire population. A “compatible” genetic model was considered the combination of an additive model in one group, with either a dominant or recessive model in the other group. Results were validated by bootstrap analysis, fixing a total number of re-sampling of 1000.

CART (Classification And Regression Tree) analysis, a recursive partitioning method, was performed to further investigate how SNPs and clinico-pathological features interact in the regulation of neoadjuvant treatment response. The process starts with the root node that contains all the complete responders (TRG = 1, *n* = 77) and the non-complete responders (TRG = 2-5, *n* = 188) subjects. At the end, the most important variables impacting the treatment response are highlighted. Terminal nodes were arbitrarily categorized in the groups based on the treatment response: low (TRG1 < 30%), medium (30% ≤ TRG1 < 60%) and high probability (TRG1 ≥ 60%) of response.

SNPs predictive of pCR were tested also for their possible prognostic value in terms of DFS, defined as the interval between surgery and relapse, death, or the last follow-up. The effect on DFS was computed by the Kaplan-Meier method, and the log-rank test was used to test differences between subgroups.

Logistic and survival analyses were performed using SAS 9.2, whereas ‘R’ statistical package version 2.6.2 was used for CART analysis.

## SUPPLEMENTARY MATERIAL FIGURE


