# Supplementary material for: Predictive role of microRNA-related genetic polymorphisms in the pathological complete response to neoadjuvant chemoradiotherapy in locally advanced rectal cancer patients
Source: Oncotarget. 2016 Feb 26;7(15):19781–93. doi: 10.18632/oncotarget.7757 (PMC4991418; doi:10.18632/oncotarget.7757)
Supplement: Supplementary file 1 [file oncotarget-07-19781-s001.pdf]

Predictive role of microRNA-related genetic polymorphisms in the pathological complete response to neoadjuvant chemoradiotherapy in locally advanced rectal cancer patients

Supplementary Material

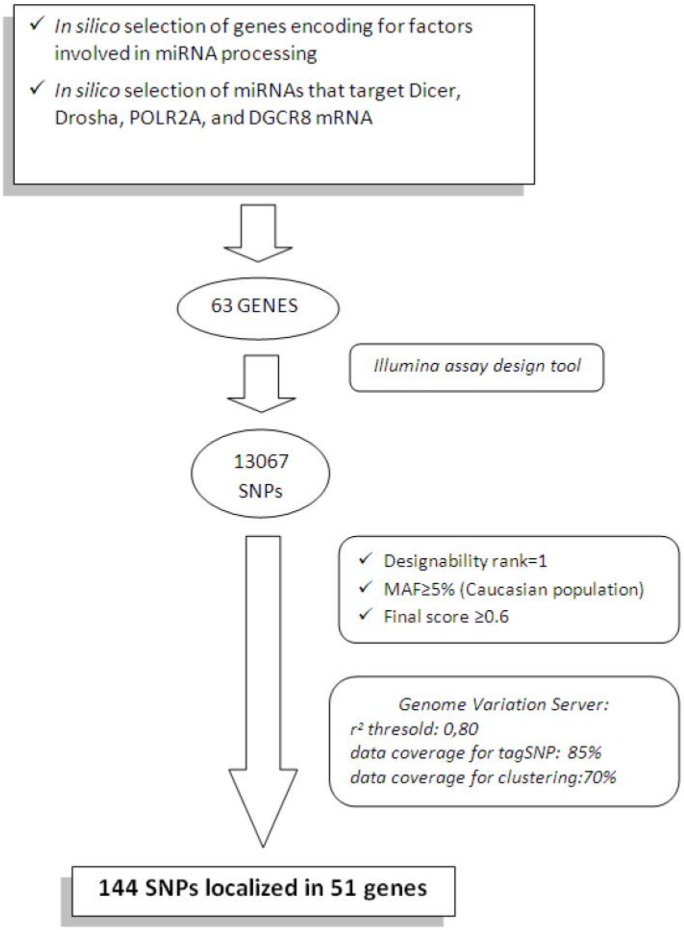

Supplementary Figure 1: Flow chart of SNPs selection.

SNP, single nucleotide polymorphism; MAF, minor allele frequency.
